# Not all shellfish "allergy" is allergy!

**DOI:** 10.1186/2045-7022-1-3

**Published:** 2011-06-10

**Authors:** Chee K Woo, Sami L Bahna

**Affiliations:** 1 Allergy and Immunology Section, Louisiana State University Health Sciences Center, Shreveport, Louisiana 71130, USA

## Abstract

The popularity of shellfish has been increasing worldwide, with a consequent increase in adverse reactions that can be allergic or toxic. The approximate prevalence of shellfish allergy is estimated at 0.5-2.5% of the general population, depending on degree of consumption by age and geographic regions. The manifestations of shellfish allergy vary widely, but it tends to be more severe than most other food allergens.

Tropomyosin is the major allergen and is responsible for cross-reactivity between members of the shellfish family, particularly among the crustacea. Newly described allergens and subtle differences in the structures of tropomyosin between different species of shellfish could account for the discrepancy between in vitro cross-antigenicity and clinical cross-allergenicity. The diagnosis requires a thorough medical history supported by skin testing or measurement of specific IgE level, and confirmed by appropriate oral challenge testing unless the reaction was life-threatening.

Management of shellfish allergy is basically strict elimination, which in highly allergic subjects may include avoidance of touching or smelling and the availability of self-administered epinephrine. Specific immunotherapy is not currently available and requires the development of safe and effective protocols.

## Introduction

Seafood consumption has increased in popularity and frequency worldwide. The largest consumer is China, followed by Japan and United States of America. In 2009, Americans ate an average of 15.8 pounds of fish and shellfish per capita, with shrimp being the top choice at 4.1 pounds [1]. The increased production and consumption of seafood has been accompanied by increasing reports of adverse reactions to seafood. Such reactions can be immune-mediated allergic reactions or non-immunologic, with both presenting with similar symptoms.

### Prevalence and epidemiology

Shellfish is one of the leading causes of food allergy in adults and is a common cause of food-induced anaphylaxis. In an international survey using a questionnaire administered to 17,280 adults (aged 20-44 years) from 15 countries, symptoms related to seafood were reported to be caused by shrimp in 2.3%, oyster in 2.3%, and fish in 2.2% [2]. In the United States, a telephone survey of 14,948 individuals revealed that 2-3% believed to have seafood allergy: 2.2% to shellfish and 0.6% to fish [3]. Shellfish allergy was much lower in children than in adults (0.5 vs 2.5%). In a decreasing frequency, the causative types of shellfish were shrimp, crab, lobster, clam, oyster and mussel.

The prevalence of shellfish allergy in Asian countries is higher than in western countries [4], and this might reflect the geographic consumption of shellfish. In a study of children residing in Singapore [5], the prevalence of shellfish allergy was more common in native children (4-6 years, 1.19%; 14-16 years, 5.23%) compared to expatriate children (4-6 years, 0.55%; 14-16 years, 0.96%). Specific shellfish allergy can reflect regional consumption of that particular species.

Only a few studies evaluated the natural history of shellfish allergy, and they seem to indicate that it is long-lasting [6]. In a study of 11 subjects with shrimp hypersensitivity [7], shrimp-specific IgE levels in all subjects were relatively constant during the 24 months of the study and were not affected by shrimp challenge. Another study [8] however, revealed that children with shrimp allergy have higher specific IgE antibody levels, show more intense binding to shrimp peptides, and a greater epitope diversity than in adults, suggesting that sensitization to shrimp might decrease by age.

### Classification of shellfish (Figure 1)

**Figure 1:**
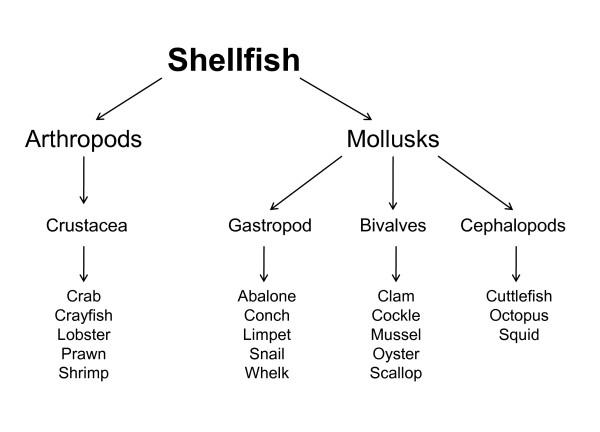
**Classification of shellfish**.

"Shellfish" and "seafood" are often used by the public interchangeably and with different meanings. "Seafood" is a general term that refers to any edible aquatic animals, whereas "shellfish" refers to those with a shell or shell-like exoskeleton i.e. crustacea and mollusks. Crustacea are classified among arthropods together with arachnids and insects, whereas mollusks include Bivalves, Gastropods and Cephalopods.

### Shellfish toxic syndromes (Table 1)

**Table 1:** Shellfish toxic syndromes

- Paralytic shellfish poisoning
- Neurotoxic shellfish poisoning
- Diarrhetic shellfish poisoning
- Amnesic shellfish poisoning
- Azaspiracids shellfish poisoning
- Adverse reactions to bacterial or viral contamination
- *Vibrio vulnificus*
- *Clostridium botulinum*
- Staphylococcal enterotoxin
- Norwalk virus

Shellfish poisoning frequently masquerade as an allergic reaction. Ingestion of contaminated shellfish results in a wide variety of symptoms depending on the concentration of toxins and amount consumed. Five types of shellfish poisoning have been identified [9]. Scombroid poisoning [10] has been linked to fish by the action of bacteria on muscle histidine and production of histamine. To the best of our knowledge, we did not encounter any reports in the English literature on scombroid poisoning from shellfish consumption.

### Paralytic shellfish poisoning

Paralytic shellfish poisoning [11] is the best known and it is caused by saxitoxins. It is the most severe, with neurological symptoms predominating. Within 30 minutes of ingestion, the first and most consistent presentation is numbness, tingling or burning of lips, tongue and throat. Paresthesias involve the face and neck and often spread to other parts of the body. Muscular weakness then affects the extremities, and in more severe cases, dysphonia, dysphagia and ataxia occur. Paralysis may occur within 2-12 hours and may persist for as long as 72 hours. Bivalve mollusks such as mussels, clams and oysters assimilate and temporarily store saxitoxins, a complex of neurotoxins produced by dinoflagellates. In the United States, paralytic shellfish poisoning is a problem primarily in the New England states on the East Coast and in Alaska, California and Washington on the West Coast. It has also been reported in Asia, Africa, Europe, Oceania and South America.

### Neurotoxic shellfish poisoning

Neurotoxic shellfish poisoning [11] is characterized by both gastrointestinal and neurologic symptoms. It resembles a mild case of paralytic shellfish poisoning but without paralysis. The onset occurs within 3 hours of ingestion of shellfish contaminated with brevetoxins. Symptoms include numbness of lips, tongue and throat which then spread to other parts of the body. Muscular aches, dizziness, reversal of hot and cold temperature sensation occur along with nausea, vomiting, abdominal pain and diarrhea. *Karenia brevis *is the dinoflagellate that synthesizes brevetoxins, a group of related heat-stable toxins that are responsible for the clinical manifestations of neurotoxic shellfish poisoning. In the United States, the illness is generally associated with the consumption of shellfish harvested along the coast of Gulf of Mexico from Florida to Texas and sporadically along the southern Atlantic coast. *K. Brevis *blooms are also known as "red tides" because of the red coloration of seawater. Unlike other shellfish toxins, the brevetoxins can aerosolize by surf and wave action along the beach during red tides. These irritant toxin aerosols can cause conjunctival irritation, sneezing and rhinorrhea that resemble an allergic response. Exposure to such toxins in individuals with underlying asthma or chronic obstructive pulmonary disease can lead to shortness of breath, non productive cough and wheezing.

### Amnesic shellfish poisoning

Amnesic shellfish poisoning [9] presents initially with vomiting, diarrhea and abdominal cramping within 24 hours after ingestion of shellfish contaminated with domoic acid. In some cases, varying degrees of neurological dysfunction ensues within 48 hours, including confusion, loss of memory and disorientation. The loss of short term memory is unique to this type of shellfish poisoning. Other neurological symptoms are headache, hyporeflexia, hemiparesis, ophthalmoplegia and altered consciousness ranging from agitation to coma, seizures and myoclonus, especially affecting the face. The phytoplanktonic diatom *Pseudo-nitzchia multiseries *are the source of the toxic agent identified as domoic acid. This potent neurotoxin accumulates in mussels and clams that feed on toxic plankton during their bloom.

### Diarrhetic shellfish poisoning

Diarrhetic shellfish poisoning [9] is the mildest and most benign of the toxic shellfish poisonings. Clinical features are limited to the GI tract and include diarrhea, nausea, vomiting, abdominal pain and cramps. Chills, fever or headache may be present in up to 10% of cases. These symptoms usually manifest in a period ranging from 30 mins to 6 hours after ingestion of contaminated shellfish. Patients often do not seek medical attention due to the transient nature of the illness and its spontaneous resolution. Diarrhetic shellfish poisoning is associated with the consumption of mussels, scallops, clams and oysters contaminated with biotoxins produced by toxic marine dinoflagellates during their blooms in summer.

### Azaspiracids shellfish poisoning

Azaspiracids (AZA) [12] are polyether marine toxins that accumulate in various shellfish species and have been associated with severe gastrointestinal human intoxications. The first confirmed case was in 1995 in the Netherlands. This toxin has since been reported in Western Europe, Northwest Africa and Eastern Canada. There have been several attempts to identify the AZA producing organism(s) and the polyether structure of these compounds might suggest a dinoflagellate origin. Unlike many of the other well-described marine phycotoxins, relatively little is known about AZA. Similar to diarhetic shellfish toxins, human consumption of AZA-contaminated shellfish can result in severe acute symptoms that include nausea, vomiting, diarrhea, and stomach cramps that persist for 2-3 days. Toxicology studies have shown that AZA can induce widespread organ damage in mice and that they can be a potent toxin.

### Adverse reactions to shellfish from bacterial and viral etiologies

In addition to the above specific five types of shellfish toxicity, the differential diagnosis should include bacterial toxins, viral and bacterial infections. Although rare, *Vibrio vulnificus *is the leading cause death related to seafood consumption in the United States. This bacterium is part of the natural flora of coastal environments worldwide and has been isolated in a variety of seafood including shrimp, fish, oysters and clams. Consumption of undercooked or raw seafood (primarily raw oysters) contaminated with *V. vulnificus *can result in severe fulminant sepsis and development of severe cellulitis with ecchymoses and bullae. Risk factors include immunocompromised conditions especially alcoholic liver disease, hepatitis B or hepatitis C, and male gender. Treatment includes antibiotics and supportive care.

Food-borne botulism occurs upon ingestion of food contaminated by preformed toxin that is produced by *Clostridium botulinum*. Initial manifestations are GI symptoms such as nausea, vomiting and diarrhea. Other initial symptoms include dry mouth, diplopia, blurred vision and photophobia caused by loss of pupillary light reflex. A symmetric descending flaccid paralysis may occur that can lead to respiratory failure.

Staphylococcal enterotoxin is produced by *Staphylococcal aureus *growing in the contaminated food. Onset of symptoms is usually rapid after ingestion. GI symptoms predominate with nausea, vomiting, abdominal cramps and diarrhea. This type of food poisoning usually occurs in foods that have been left at room temperature for some time.

Norwalk virus infection usually occurs after ingestion of contaminated raw shellfish and can spread through the fecal-oral route. Incubation period is 24-48 hours after exposure. Most common symptoms are nausea, vomiting and diarrhea which resolve after 24 hours.

### Shellfish toxicity recognition and management

Shellfish poisoning may be under diagnosed particularly when mild, or misdiagnosed as allergy. The presence of similar symptoms in other individuals who shared the same meal, absence of prior reactions to the same shellfish and its subsequent tolerance without symptoms should favor toxicity. The level of suspicion should be higher in regions with seasonal algal blooms, high levels of biotoxins or toxic algae. In the majority of these toxic syndromes, the toxin does not alter the taste and appearance of the shellfish and is not inactivated by usual cooking.

Treatment for these toxic syndromes is mostly supportive with respiratory support in cases where neurological involvement can cause respiratory failure. In acute severe cases, gastric emptying and administration of activated charcoal has been recommended to help block further absorption of the toxins.

### Shellfish allergens (Table 2) [13]

**Table 2:** Allergenic proteins characterized in shellfish and other invertebrates

Group	Tropomyosin	Arginine kinase	Myosin light chain	Sarcoplasmic binding protein
**Crustacea**	Crab (Cha f 1) North Sea shrimp (Cra c 1) American lobster (Hom a 1) Whiteleg shrimp (Lit v 1) Shrimp (Pen a 1, Pen i 1) Spiny lobster (Pen s 1) Black tiger shrimp (Pen m 1)	North Sea shrimp (Cra c 2) Whiteleg shrimp (Lit v 2) Black tiger shrimp (Pen m 2)	Brine shrimp (Art fr 5) North Sea shrimp (Cra c 5) American lobster (Hom a 3) Whiteleg shrimp (Lit v 3) Black tiger shrimp (Pen m 3)	North Sea shrimp (Cra c 4) Whiteleg shrimp (Lit v 4) Black tiger shrimp (Pen m 4) Narrow-clawed crayfish (Pon l 4)

**Mollusca**	Pacific oyster (Cra g 1) Abalone (Hal d 1) Brown garden snail (Hel as 1) Scallop (Mim n 1) Tropical green mussel (Per v 1) Squid (Tod p 1)	--	--	--

**Other invertebrates**	Anisakis simplex (Ani s 3) Common roundworm (Asc l 3) German cockroach (Bla g 7) Blomia Tropicalis mite (Blo t 10) Midge (Chi k 10) House dust mite (Der f 10, Der p 10) Storage mite (Lep d 10, Tyr p 10) Silverfish (Lep s 1) American cockroach (Per a 7)	Silk moth (Bomb m 1) House dust mite (Der p 20) American cockroach (Per a 9) Indianmeal moth (Plo i 1)	German cockroach (Bla g 8)	--

Hoffman et al [14] first isolated two allergens from raw and cooked shrimp, termed antigen I and antigen II respectively. The heat stable antigen II, demonstrated specific IgE binding in the sera of all 11 shrimp-allergic subjects tested. Subsequently, other studies confirmed that antigen II is the major shrimp allergen and was identified as tropomyosin [15-17]. The latter belongs to a family of proteins associated with the thin filament in muscle cells and microfilaments in non-muscle cells. Tropomyosin is not only a major crustacean allergen, it has also been demonstrated in a number of mollusk species [18]. In contrast to invertebrate tropomyosin, vertebrate tropomyosins are not allergenic. Dot blot and immunoblot analysis on subjects with a history of meat allergy to vertebrate meats did not show any IgE binding to tropomyosin of beef, pork, rabbit or chicken [19,20]. Similar studies demonstrated that shrimp allergic subjects' specific IgE did not cross react with any mammalian tropomyosins or their fragments [18,21].

Tropomyosin is heat stable [16], yet its allergenicity may change by certain processing methods. Boiling may result in the Maillard reaction (glycation) and formation of neoepitopes [22], as demonstrated that in some patients, boiled shrimp extract induced larger skin test responses than raw extract [23]. Also, shrimp extract treated with high intensity ultrasound for 180 minutes demonstrated decreased binding with sera from shrimp allergic patients [24].

In addition to tropomyosin, other allergens have been identified and characterized in shellfish. Arginine kinase a potential new class of invertebrate pan-allergens have been identified in Pacific white shrimp and Black tiger prawn as Lit v 2 and Pen m 2 respectively [25,26]. Arginine kinase has also been found in mollusks [27], along with other allergens such as myosin heavy chain, haemocyanin, and amylase [28]. However, the clinical significance of these allergens in mollusks is currently undefined.

Two other allergens identified in the Pacific white shrimp (*Litopenaeus vannamei*) are myosin light chain kinase [29] and sarcoplasmic calcium binding protein [30], identified as Lit v 3 and Lit v 4 respectively. Sarcoplasmic calcium binding protein seems to be an important allergen in the pediatric population, recombinant sarcoplasmic calcium binding protein was recognized by serum IgE from 20 of 52 (38.4%) shrimp allergic subjects, with a higher frequency in children (17 out of 23; 74%) than in adults (3 out of 29; 10%) [30].

### Cross-reactivity within the shellfish family

Subjects with shrimp hypersensitivity usually clinically react to other types of crustacea. Tropomyosin showed very high homologies of up to 98% among crustacean species, including crawfish, crab and lobster [17,31,32]. Crustacean allergic subjects also often react to species of the mollusk group. Leung et al [18] demonstrated in vitro that sera from nine crustacean allergic patients had IgE binding to antigens from all 10 mollusk species tested. However, in vitro cross-antigenicity does not necessarily indicate clinical cross allergenicity.

Furthermore, Jirapongsananuruk et al [33] demonstrated that shrimp allergy can be species-specific. Some studies reported clinical reactivity of 38% between shrimp and other crustacean members, 14% between crustacea and mollusks, and 49% between mollusk members [3,18,34,35]. It should be noted that these figures were derived primarily from self-reported clinical reactions.

### Cross-reactivity with other invertebrate antigens

In patients with respiratory allergies, house dust mite (HDM) tropomyosin has been demonstrated to be a major allergen [36]. Tropomyosin from *D. pteronyssinus *(Der p 10) has homology of 75-80% to shrimp and fruitfly and 65% to mollusks [37]. Tropomyosins from HDM and cockroach have high sequence identities to shellfish tropomyosin of around 80% [38,39]. Such data indicate possible sensitization to tropomyosin by inhalation from a variety of non-crustacean sources [40], hence the emerging terminology "mite-crustaceans-mollusk syndrome".

In a study of 9 Orthodox Jews, who observe Kosher dietary laws that prohibit eating shellfish, the presence of IgE sensitization to shrimp was explored [41]. All 9 subjects had perennial respiratory allergies and positive HDM skin test, which was also positive to shrimp in all 9 and to cockroach in 2 of 7 tested.

High levels of serum IgE to tropomyosin is correlated with severity of shellfish allergy, however it may not be the only allergen responsible for shellfish sensitization in HDM sensitized individuals [42]. Sera from patients with shrimp allergy and HDM sensitivity but not reactive to tropomyosin were studied and found to cross react with a new 20-kDa allergen present in both shrimp and HDM [43]. The authors postulated that this allergen could correspond to the sarcoplasmic calcium-binding protein and myosin light chain allergens. Arginine-Kinase found in decapod crustaceans and HDM (Der p 20) have also been proposed as a probable pan-allergen with 78% sequence homology with shrimp allergen Pen m 2 [44].

Some reports suggested that HDM injection immunotherapy may enhance sensitization or worsening allergy to shellfish [45-48]. However, a prospective study was conducted by Asero [49] on non shrimp-sensitized subjects receiving HDM injection immunotherapy for respiratory allergy and allowed to eat shellfish. After 3 years of HDM immunotherapy, participants showed no reactions to shrimp by skin testing or open oral challenge. Therefore, there is no strong evidence that shellfish allergy can develop through HDM immunotherapy.

It should be noted that crustacean and mollusk allergens do not cross-react with fish allergens and no reactivity between known allergens or homologous proteins have been demonstrated [50]. However, patients who are allergic to *Anisakis simplex *may react to parasitized fish or shellfish. Although tropomyosin might not be the major allergen in Anisakis allergy [51,52], possible clinical cross reactivity might occur in crustacean allergic subjects due to a high (74%) amino acid sequence homology of crustacean and Anisakis tropomyosin [50,53-55].

### Clinical presentations of shellfish allergy

Symptoms of shellfish allergy can range from mild urticaria to life threatening anaphylaxis. Most reactions are IgE-mediated with rapid onset and may be gastrointestinal, cutaneous, or respiratory. Symptoms may be limited to transient oral itching or burning sensation (oral allergy syndrome) within minutes of eating shellfish [50]. Recently, a case of food protein-induced enterocolitis syndrome to shellfish has been reported in a 6-year-old boy after ingestion of clam [56]. Food-dependent exercise-induced anaphylaxis has also been described to shellfish [57,58]. In certain subjects, anaphylaxis may result by a synergistic effect of nonsteroidal anti-inflammatory drugs with shellfish intake [59].

Shellfish protein is a potent allergen and can provoke symptoms by inhalation or skin contact [60]. Airborne allergens are particularly abundant in the vicinity of cooking shellfish by boiling, steaming, or frying. Occupational exposure, such as in snow crab processing plants, not only can cause symptoms in highly allergic subjects, but can also cause de novo sensitization [61]. Symptoms may be limited to the respiratory tract or affect other systems such as the skin or systemic anaphylaxis. The reported estimate of prevalence of occupational asthma in shellfish-processing workers is 2-36% [62].

Exposure by skin contact occurs more in occupational settings and the manifestations are commonly cutaneous in the form of urticaria or allergic contact dermatitis, although occasionally it can be systemic. The prevalence of occupational contact dermatitis to shellfish ranged from 3 to 11% [62]. At least one case of contact urticaria to shrimp was reported to be caused by the protein in the shell and not to the meat itself [63].

### Diagnostic approach

It is important at the outset to establish whether the adverse reaction is caused by shellfish allergy or toxicity. A detailed history is essential, with emphasis on the specific implicated type of seafood, the amount eaten, the type of symptoms, time of onset, and symptoms in other individuals who consumed the same meal. Management of food poisoning is mostly symptomatic.

In addition to the history and type of manifestation, allergic reactions are supported by documenting sensitization, i.e., positive skin test or elevated specific IgE level. Unless the patient had a life-threatening reaction, verification by a titrated oral challenge should be performed, preferably in a blind, placebo-controlled manner [64,65]. It is worth noting that skin testing with commercial extracts may give a false negative result whereas the prick-to-prick method can be more reliable, particularly by using the same implicated food. Carnes et al [23] demonstrated that using cooked instead of raw extracts for skin prick testing correlated well with food challenge.

If the medical history reveals concomitant factors, such as exercise, alcohol ingestion, or intake of nonsteroidal anti-inflammatory drugs, such factor(s) should be incorporated in the challenge test.

### Management

In general, management of any food allergy is basically strict avoidance based on proven clinical reactions and not mere sensitization. Because of cross-reactivity, avoidance of all crustacea is generally advised. Avoidance of mollusks is not necessary unless the patient is concomitantly allergic to it. Since cross-reactivity among crustacea is not complete, it is possible that allergy be limited to certain crustacea members. Patients should be alerted to possible inadvertent hidden exposure to the offending food, particularly in restaurants where cooking equipment or serving utensils may be used for different foods.

In addition to strict avoidance, patients who have had severe reactions should be advised to wear a Medic Alert identification and be trained in using epinephrine autoinjector. Because future reactions may be more severe [66,67], some physicians tend to prescribe epinephrine autoinjectors to most food-allergic subjects.

Although there have been some recent investigational protocols for oral or sublingual immunotherapy to certain foods, to the best of our knowledge none has been done with shellfish. Perhaps some reports will be seen in the near future.

## Conclusion

Shellfish is one of the major food allergens and its consumption is increasing worldwide. It is important to distinguish between shellfish allergy and toxicity as their presentations can mimic each other. Tropomyosin has been identified as the major allergen in the shellfish family and is responsible for the majority of the cross-reactivity observed. However, newly discovered allergens and subtle differences in the structures of tropomyosin between different species of shellfish could account for the differences between cross-reactivity and true clinical reactions. Further research is needed to identify allergens with improved clinical sensitivity and species specificity which would aid in diagnosis. The primary management of shellfish allergy is avoidance. Patients who have immunodeficiency should only eat cooked shellfish due to the risk of severe infections from ingesting raw shellfish contaminated with infectious organisms.

## Competing interests

The authors declare that they have no competing interests.

## Authors' contributions

CKW collected and reviewed the literature and shared in preparing the manuscript. SLB shared in preparing the manuscript. All authors read and approved the final manuscript.
